# ^18^F-FDG PET/CT for Oncological Patients: Procedural Guideline by the Korean Society of Nuclear Medicine Version 2.0

**DOI:** 10.1007/s13139-025-00928-y

**Published:** 2025-07-17

**Authors:** Eun Ji Han, Chae Hong Lim, Jinkyoung Oh, Joon Young Choi, The Korean Society of Nuclear Medicine Medical Affairs Committee

**Affiliations:** 1 Department of Nuclear Medicine, Yeouido St. Mary’s Hospital, College of Medicine, The Catholic University of Korea, Seoul, Republic of Korea; 2 Department of Nuclear Medicine, Soonchunhyang University Seoul Hospital, Seoul, Republic of Korea; 3 Department of Nuclear Medicine, Incheon St. Mary’s Hospital, College of Medicine, The Catholic University of Korea, Seoul, Republic of Korea; 4 Department of Nuclear Medicine, Samsung Medical Center, Sungkyunkwan University School of Medicine, 81 Irwon-ro, Gangnam-gu, Seoul, 06351 Republic of Korea

**Keywords:** ^18^F-FDG, PET/CT, Oncology, Procedural Guideline

## Abstract

The Korean Society of Nuclear Medicine (KSNM) was founded in 1961 to promote the clinical and technical advancement of nuclear medicine in Republic of Korea. It comprises approximately 600 members, mainly nuclear medicine physicians and related scientists. The KSNM periodically updates guidelines to advance nuclear medicine and help medical professionals provide better patient care. These guidelines are flexible and not obligatory. The KSNM states that these guidelines should not be used in legal actions challenging the medical decisions of healthcare professionals. Final medical decisions should be made by nuclear medicine physicians based on individual patient conditions, available resources, the latest medical knowledge, and technological advances. Deviations from these guidelines does not necessarily indicate substandard care, but rather reflects the application of reasonable clinical judgment. Detailed quality control is to follow the KSNM’s quality control guidelines. Due to the diversity of patients and complexity of medical cases, adherence to guidelines does not always guarantee accurate diagnoses or successful outcomes. This guideline aims to revise the 2013 ‘^18^F-fluorodeoxyglucose Positron Emission Tomography/Computed Tomography for Oncological Patients: Procedural Guideline by the KSNM’, ensuring medical professionals take appropriate actions based on current medical knowledge, available resources, and patient needs, ultimately achieving effective and safe imaging.

**Clinical Trial Number**: Not applicable.

## Introduction

^18^F-fluorodeoxyglucose (^18^F-FDG) positron emission tomography (PET)/computed tomography (CT) is an imaging technique for visualizing tissue glucose metabolism, widely used for diagnosing, staging, identifying recurrences, and assessing treatment effectiveness in various kinds of malignant tumors. This guideline has been modified and reviewed by experts, considering the guidelines by the ‘Society of Nuclear Medicine and Molecular Imaging (SNMMI)’ and ‘European Association of Nuclear Medicine and Molecular Imaging (EANMMI)’, and the current medical evidence related to ^18^F-FDG PET/CT in tumors, to fit the context of Republic of Korea [1, 2]. The guideline includes clinically applicable general information such as clinical indications for examination, procedures, image interpretation, and performance and quality control of imaging equipment. Additionally, it addresses the implementation of ^18^F-FDG PET/CT examinations in pediatric tumor patients, a topic not covered in previous version of guideline.

Although the ^18^F-FDG PET/CT examination is playing an increasingly important role in imaging inflammation, infection, cardiology, and neurology, these fields are not included in this guideline.

## Goals

This guideline provides general information on the clinical indications, imaging procedures, and interpretation of ^18^F-FDG PET/CT examinations in tumors. It aims to offer practical assistance to nuclear medicine healthcare providers and staff conducting examinations, enhancing the quality of examinations and their appropriate implementation. Its ultimate goal is to provide suitable nuclear medicine technology for oncological patients, to improve quality of life and health, and to contribute to the effective use of healthcare resources. As medicine is a continually evolving field, the contents of these guidelines are intended to serve as reference materials reflecting the current point in time and not as unchangeable directives. In addition, information related to the ^18^F-FDG PET/CT procedures in Republic of Korea will also be provided based on a survey conducted in June 2024, covering 79 PET/CT scanners across 61 institutions.

## Definitions

### PET

An imaging technique that involves injecting a radiopharmaceutical that emits positrons into the body, detecting the resulting 511 keV gamma radiation using a PET scanner, and reconstructing images to visualize the distribution of the injected radiopharmaceutical within the body.

### PET/CT Scanner

Equipment that acquires both PET and CT images; the CT images are utilized to correct for attenuation of 511 keV gamma radiation and scattering of the PET images, and to provide anatomical imaging information with high spatial resolution. By obtaining fused images of PET and CT, accurate information on the location or morphology of lesions, which may be difficult to ascertain with PET images alone, can be used in more accurate diagnoses.

### PET/CT Image Acquisition Coverage

The range of image acquisition can be altered to suit specific purposes.

1. Whole body imaging: imaging that includes from the skull vertex to the toes
2. Torso imaging: imaging that includes from the skull base to the mid-thigh
3. Regional imaging: imaging that captures only a localized area (e.g. obtaining images only from the thorax or head and neck)
4. Additional imaging: acquiring delayed images or images from other areas following whole body or torso imaging

## Clinical Indications

### Adult Patients

^18^F-FDG PET/CT helps determine the appropriate medical care of oncological patients. ^18^F-FDG PET/CT is performed when there is a reasonable expectation that it will have an impact on the patient’s treatment. The avidity of ^18^F-FDG varies with the tumor type. Therefore, if ^18^F-FDG PET/CT is performed in cases with insufficient evidence of efficacy, it is recommended that the patient be informed of the benefits and potential risks associated with the examination. Common indications for ^18^F-FDG PET/CT in adult oncology include the following:

1. Differentiation between benign and malignant lesions
2. Staging in patients with known malignancies
3. Evaluation of response to treatment for known malignancies
4. Differentiating whether residual abnormalities detected on other imaging studies after treatment are tumor or treatment-related change (inflammation, fibrosis, or necrosis)
5. Detection of unknown primary cancer when metastatic disease is detected as the first
6. Detection of hidden malignancies, especially in the presence of elevated tumor markers
7. Radiation therapy planning
8. Selection of the tumor sites expected to provide the best diagnostic information for biopsy

The clinical indications for ^18^F-FDG PET/CT are not limited to the above list and it is not possible to provide a complete list of indications, because the clinical utility of ^18^F-FDG PET/CT in oncology is constantly expanding. In addition, in Republic of Korea, ^18^F-FDG PET/CT is to be performed in cancer patients according to the national health insurance reimbursement guideline, so it is necessary to check the guideline before the examination.

### Pediatric Patients

The clinical indications for ^18^F-FDG PET/CT in pediatric oncology are not significantly different from those in adults, and common indications include the followings:

1. Lymphoma: initial staging, assessment of response to treatment, restaging, radiation therapy planning, and providing prognostic information [3–6]
2. Sarcoma (osteosarcoma, Ewing sarcoma, rhabdomyosarcoma, and other soft tissue sarcomas): initial staging, assessment of response to treatment, restaging, planning of radiation therapy, and providing prognostic information [7–13]
3. Neuroblastoma: providing prognostic information [14–17]
4. Central nervous system tumor: tumor grading, providing prognostic information, and differentiation between viable tumor and post-radiotherapeutic change [18–20]
5. Head and neck cancer [21]
6. Langerhans cell histiocytosis [22–24]
7. Post-transplant lymphoproliferative disorder [25]
8. Germ cell tumor: staging, detection of relapse [26]
9. Wilms tumor [27, 28]
10. Neurofibromatosis type 1: when malignant transformation of neurofibroma is suspected [29, 30]

## Qualifications and Responsibilities of Personnel

Personnel must comply with the technical standards of the Korean Society of Nuclear Medicine (KSNM) for diagnostic procedures using radiopharmaceuticals [31].

## Procedure/Specifications of the Examination

### Radiopharmaceuticals

^18^F-FDG is the radiopharmaceutical most commonly used in oncologic PET. The radionuclide ^18^F is produced in a cyclotron and has a half-life of approximately 110 min. ^18^F-FDG is a glucose analog absorbed into cells via glucose transporters and incorporated into the first step of the physiological glucose metabolic pathway. Consequently, the degree of ^18^F-FDG uptake reflects the metabolic activity of cells [32]. Although other radiopharmaceuticals have also been used for oncologic PET, this procedural guideline is limited to ^18^F-FDG PET/CT only. This guideline does not address the use of other radiopharmaceuticals for PET or the use of brain PET and PET/MRI.

The recommended dose of ^18^F-FDG for adults is 2.5–5.0 MBq/kg (± 10%), equating to 150–300 MBq for a 60 kg adult. In Republic of Korea, a survey of domestic institutions showed that the average dose administered to adults was 4.0 MBq/kg (median: 3.7 MBq/kg; range: 1.9–5.2 MBq/kg). For pediatric patients, the dose is 3.7–5.2 MBq/kg (with a minimum dose of 26 MBq or 0.7 mCi). The dose for children should be based on their body weight and kept as low as reasonably achievable while still obtaining diagnostic-quality images. The dose can be adjusted according to the specifications of the scanner or the imaging protocol. Detailed guidelines for pediatric dosing are provided in the 2016 North American Consensus Guidelines update and the EANMMI’s pediatric dose recommendations [33, 34]. According to a survey conducted in Republic of Korea, approximately 70% of the hospitals surveyed use a recommended dose of 3.7–5.2 MBq/kg, with the remaining majority using less than 3.7 MBq/kg.

The effective dose of PET varies with the administered activity and patient age. The effective dose is 0.0192 mSv/MBq for adults, 0.022 mSv/MBq for 15-year-olds, 0.0323 mSv/MBq for 10-year-olds, 0.0482 mSv/MBq for 5-year-olds, and 0.0801 mSv/MBq for 1-year-olds [35, 36]. The effective dose from CT can vary depending on whether it is a diagnostic CT or a low-dose CT, with a typical range of 1–10 mSv (or higher depending on the equipment used) [2].

### Patient Preparation and Precautions

Nuclear medicine physicians, nurses, or technicians should provide patients with detailed explanations about pre-exam precautions and procedures to ensure a safe and accurate examination [37, 38].

1. Fasting and hydration before examination

Patients should fast for at least 4 h before the ^18^F-FDG PET/CT scan and avoid oral or intravenous intake of substances containing glucose or sugar. Adequate hydration is encouraged starting 1–2 h before the scan (e.g. drinking at least 0.5 L of water or receiving intravenous fluids without glucose) to promote renal excretion of ^18^F-FDG.

2) Pregnancy and breastfeeding

If necessary, a pregnancy test should be performed before the scan. For those who are pregnant or suspected of being pregnant, the benefits and potential risks of the ^18^F-FDG PET/CT scan should be carefully considered before proceeding. Although ^18^F-FDG is minimally excreted in breast milk and does not require that breastfeeding be stopped, per the International Commission on Radiological Protection’s recommendations, it is advisable to limit close contact between the breastfeeding parent and infant for 12 h post ^18^F-FDG administration to minimize radiation exposure. It is recommended to breastfeed before the ^18^F-FDG injection and maximize the interval until the next feeding. Pumping and bottle-feeding pre-expressed breast milk can help minimize close contact.

3) Blood glucose management and precautions for diabetic patients

Blood glucose levels should be measured and recorded just before the ^18^F-FDG injection. Ideally, blood glucose levels should be < 11 mmol/L (< 200 mg/dL), but higher levels are not an absolute contraindication for the examination. For diabetic patients on insulin therapy, it is recommended to wait at least 4 h after rapid-acting insulin and at least 6 h after short-acting insulin before injecting ^18^F-FDG. Intermediate or long-acting insulin should not be administered on the day of the ^18^F-FDG PET/CT scan before the injection.

4) Medication before the examination

All medications used must be questioned and documented because they may affect the examination results. Administration of granulocyte colony-stimulating factor (G-CSF) for the treatment of chemotherapy-induced neutropenia may increase physiological ^18^F-FDG uptake in the bone marrow; therefore, an interval of at least 10 days is recommended after G-CSF administration to minimize the effect of G-CSF on the bone marrow [39]. The long acting form of G-CSF may increase physiological ^18^F-FDG uptake in the bone marrow for a longer period; caution should be exercised. Nodular ^18^F-FDG uptake may occur transiently in the axillary, supraclavicular, and lower cervical nodes for 4–6 weeks, following intramuscular vaccination in the ipsilateral deltoid [40, 41]. These findings should be particularly considered in patients with breast or head and neck cancer where axillary or low cervical node evaluation is important [42]. In diabetic patients, metformin use may increase ^18^F-FDG uptake in the gastrointestinal tract, but does not necessarily require discontinuation prior to the examination.

5) Reduction of physiological uptake

Because ^18^F-FDG uptake in normal tissues such as skeletal muscle, myocardium, and urinary system can interfere with accurate image interpretation, efforts are needed to minimize it. Patients should avoid strenuous physical activity for 24 h prior to the injection to minimize ^18^F-FDG uptake in muscles. To prevent ^18^F-FDG uptake in brown fat, the waiting area should be maintained at an appropriate room temperature and should not be too cold or windy. If necessary, beta-blockers or diazepam may be used prior to ^18^F-FDG administration to reduce ^18^F-FDG uptake in brown fat. In addition, urinating immediately before the scan can reduce urinary uptake [43].

6) Assessment of response to chemotherapy and radiation therapy

External radiation therapy can increase ^18^F-FDG uptake by causing local inflammation in the irradiated area. False positive findings can be observed up to 12 weeks or more after radiotherapy [44–47]. In addition, careful interpretation of ^18^F-FDG PET/CT is needed in patients who have undergone radiation therapy, because normal tissues included in the radiation therapy field may have decreased or increased ^18^F-FDG uptake. Bone marrow, brain, or tonsils within the irradiated area may have decreased ^18^F-FDG due to impairment of physiologic function, while soft tissues such as muscle or subcutaneous fat may have increased ^18^F-FDG uptake. Increased ^18^F-FDG uptake may also be seen in organs such as the lungs or liver due to radiation-induced pneumonia or hepatitis [48]. In patients receiving chemotherapy, it is recommended that ^18^F-FDG PET/CT for response evaluation be performed at least 3 weeks after completion of chemotherapy to minimize treatment-related inflammatory uptake [49]. Increased ^18^F-FDG uptake may be seen in some organs after chemotherapy. Red bone marrow may have diffusely increased ^18^F-FDG uptake due to normal hematopoietic recovery for several months after chemotherapy-induced bone marrow suppression [50]. Thymus and tonsils may also have increased ^18^F-FDG uptake for several months associated with physiological reaction after chemotherapy [51–53].

Immunotherapy, such as immune checkpoint inhibitors, may cause pseudoprogression (transient increase in tumor burden and/or appearance of new lesions) due to migration of immune cells into tumor sites, so careful interpretation of ^18^F-FDG PET/CT is needed [54].

7) Sedation

If necessary, ^18^F-FDG PET/CT can be performed under sedation. If sedation or anesthesia is required, institutional policy should be followed [2].

### Required Clinical Information

Prior to conducting the examination, verifying the following clinical information can aid in accurate image interpretation:

- Fasting status
- History of diabetes and blood glucose levels
- Patient’s weight and height
- Location of pathophysiological abnormalities and symptoms
- Presence of fever or elevated acute inflammatory markers (e.g. C-reactive protein or erythrocyte sedimentation rate)
- History of trauma, recent surgeries, or invasive diagnostic procedures
- History of malignancy, recent chemotherapy, or radiation therapy
- Known infections, inflammatory conditions, or immunosuppressive states
- Presence of benign diseases with high tissue proliferation
- Pregnancy status, suspected pregnancy, breastfeeding, and date of last menstruation
- Vaccination history

### Radiopharmaceutical Administration and Imaging

1. ^18^F-FDG administration
  1. The general dose recommendations for ^18^F-FDG administration in both adult and pediatric patients are provided in “Radiopharmaceuticals” section. In clinical settings, the dose may be adjusted to maintain image quality, considering factors such as patient weight, PET system parameters (e.g. PET bed overlap, detector configuration), and acquisition time per bed position.
  2. For patients weighing over 75 kg, a slightly higher dose than the calculated amount may be administered to compensate for excessive attenuation, which can reduce image quality. Alternatively, increasing the bed acquisition time can help maintain the recommended dose for overweight patients.
  3. Radiopharmaceuticals are preferably injected intravenously on the contralateral side of any known or suspected lesion.
  4. PET/CT scanning is generally commenced approximately 60 min after ^18^F-FDG administration. Patients are instructed to void immediately before imaging.

2) PET image acquisition
  1. Image acquisition and reconstruction parameters vary depending on the PET/CT system (e.g. scanner and software), clinical indications, specific requests from referring clinicians, and areas of interest. Institutional guidelines should be followed, and each facility may have its own PET/CT protocol.
  2. Images are acquired with the patient in a supine position, covering the torso from the base of the skull to the mid-thighs.
  3. If lesions are suspected outside the torso, whole-body PET/CT imaging (from vertex to feet) or additional imaging beyond the torso may be acquired. This approach is particularly useful for malignancies with systemic involvement (e.g. lymphoma) or tumors that commonly affect peripheral areas (e.g. malignant melanoma or skin cancer).
  4. PET acquisition time per bed position generally ranges from 1 to 3 min or longer in step-and-shoot mode, depending on the administered radiopharmaceutical dose, patient body weight, and PET system sensitivity (primarily determined by detector configuration and acquisition method). For scanners using continuous bed motion, acquisition duration is expressed as table speed (mm/s), and similar factors apply when determining scan parameters.
  5. Motion correction techniques, such as software-based motion correction or hardware-based methods like gated PET, can be employed to minimize artifacts caused by motion and improve PET/CT image alignment. In Republic of Korea, approximately 38% of surveyed PET/CT scanners incorporate motion correction techniques.
  6. If necessary, delayed imaging targeting 1–2 bed positions may be performed 90–120 min after ^18^F-FDG administration for further lesion evaluation [1, 55]. Delayed imaging enhances the target-to-background ratio due to differential clearance rates between tumor and normal tissue, aiding tumor assessment. However, increased noise from ^18^F decay may reduce image contrast, necessitating careful evaluation based on the lesion and purpose of the examination.

3) CT image acquisition
  1. CT imaging is performed for attenuation correction and anatomical localization.
  2. Various CT acquisition parameters (e.g. tube current, voltage, slice thickness, rotation time, and pitch) can be adjusted to optimize image quality while minimizing radiation exposure.
  3. To reduce radiation exposure, dose-reduction techniques such as automatic dose modulation, patient size-adjusted tube voltage selection, and iterative reconstruction algorithms are recommended where possible. In Republic of Korea, 96% (*n* = 76) of the 79 surveyed PET/CT scanners incorporate CT dose-reduction programs.
  4. Contrast-enhanced CT may be performed if clinically indicated. Prior to contrast administration, allergy history and renal function (e.g. glomerular filtration rate, serum creatinine levels) should be considered.

### Image Processing

Initial data acquired from PET scanners must be reconstructed to accurately depict the distribution of radiopharmaceuticals in tissues and organs. Data obtained in 3D mode can be reconstructed using 3D algorithms directly or converted into 2D data for 2D reconstruction.

Reconstruction algorithms include iterative reconstruction techniques such as Ordered-Subset Expectation Maximization (OSEM) or the Filtered Back Projection (FBP) method with iterative methods preferred for lower noise and higher sensitivity. FBP may be used when iterative reconstruction produces images suspected of containing quantitative errors.

During image reconstruction, corrections for attenuation, scatter, random events, and dead time, as well as detector sensitivity normalization, must be applied for accurate quantitative analysis. To quantify ^18^F-FDG uptake, the standardized uptake value (SUV) can be calculated using local radioactivity concentration and patient parameters such as body weight, lean body mass, or body surface area.

Advanced techniques such as Time-of-Flight (TOF) enhance PET scan accuracy and image quality [56]. The Point-Spread Function (PSF) reconstruction algorithm improves isotropic spatial resolution, reduces spill-in/spill-out artifacts, and enhances lesion detectability by increasing SUV or radiotracer concentration (Bq/mL) [56]. In Republic of Korea, TOF is utilized in 78% (*n* = 62) and PSF in 68% (*n* = 54) of surveyed PET/CT systems.

## Image Interpretation and Reporting

### Physiologic ^18^F-FDG Distribution

At 60 min post injection, physiologic ^18^F-FDG uptake is typically observed in the brain, heart, kidneys, and urinary tract [57, 58]. Myocardial uptake is generally low in fasting conditions, as the myocardium primarily utilizes free fatty acids instead of glucose; however, uptake can be elevated in some patients. Muscle uptake is influenced by recent physical activity and insulin administration. Gastrointestinal uptake varies among patients and is commonly observed in those taking metformin [59]. Increased uptake may be seen in Waldeyer’s ring and peripheral lymphoid tissues. Thymic uptake is occasionally observed in pediatric and adolescent patients [60]. Brown adipose tissue uptake is more frequently seen in younger patients and in cold environments [61]. Increased bone marrow uptake can be seen in patients with anemia or inflammation. After chemotherapy or G-CSF administration, generalized uptake in the bone marrow may be observed [62].

### Image Interpretation

1. Technical conditions of PET/CT imaging

PET/CT imaging comprises several key components: (a) PET images, which reflect metabolic activity in the body based on the uptake of radiopharmaceuticals; (b) CT images, which provide anatomical structures; (c) PET/CT fusion images, integrating metabolic activity with anatomical details; and (d) Maximum Intensity Projection (MIP) images, which reconstruct 3D representations from 2D PET images by selecting the maximum signal intensity along projection lines.

PET images require pixel depths of at least 16 bits to ensure accurate value representation and are expected to utilize appropriate image scaling and color mapping. These images are recommended to be presented in the axial plane while maintaining alignment with coronal and sagittal morphological views. When transmitting these images to Picture Archiving and Communication System (PACS), adherence to Digital Imaging and Communications in Medicine (DICOM) standards is essential. PET axial, CT axial, PET/CT fusion axial, and MIP images should be included in PACS transmissions to facilitate comparisons. The upper and lower SUV window limits for PET/CT fusion and MIP images are recommended to remain consistent across images for better comparability.

A survey for the institutions in Republic of Korea showed that all 61 participating centers uploaded PET/CT fusion axial and MIP images to PACS, with PET axial images being uploaded by 97% (*n* = 59) and CT axial images by 98% (*n* = 60). Regarding SUV window settings for MIP images, ranges of 0–7 (28%), 0–10 (21%), and 0–5 (15%) were most common. Similarly, PET/CT fusion images predominantly used SUV window settings of 0–7 (26%) and 0–10 (26%), followed by 0–5 (15%).

2) Visual interpretation

The uptake patterns of ^18^F-FDG are visually analyzed and categorized based on their configuration, such as focal, linear, or diffuse, and their intensity is classified as mild, intermediate, or intense, distinguishing physiological uptake from pathological uptake. PET findings should be interpreted in correlation with CT’s anatomical information.

3) Quantitative analysis
  1. Various quantitative methods

Various quantitative methods are employed for PET/CT analysis:

- Maximum SUV (SUVmax)/SUV normalized by lean body mass (SULmax): The highest SUV value measured within a single voxel of the lesion.
- SUVpeak/SULpeak: The average uptake in a 3D spherical volume-of-interest (VOI) of 1.2 cm diameter (1.0 mL volume) positioned to maximize the value.
- Mean SUV (SUVmean), Metabolic Tumor Volume (MTV), and Total Lesion Glycolysis (TLG): The SUVmean represents the average uptake within a VOI, MTV reflects the VOI’s volume, and TLG is calculated by multiplying SUVmean by MTV.
  (2) VOI boundary delineation methods

Three main methods for VOI boundary delineation include:

- Using fixed SUV or SUL thresholds (e.g. 2.5 or 3.5), where voxels with uptake values exceeding a predefined absolute cutoff are included in the VOI [63].
- Defining boundaries based on a percentage of SUVmax or SULmax, where voxels with uptake ≥ a certain percentage of the maximum uptake within the lesion are included (e.g. 50% of the maximum voxel value, VOI50%) [64, 65].
- Employing gradient-based methods, which identify the boundary at the point of greatest change in uptake between the lesion and surrounding tissue [66].
  (3) Texture analysis

Texture analysis quantifies metabolic heterogeneity within a tumor by evaluating the spatial distribution of uptake within the VOI. Although it is not yet routinely applied in clinical practice, it is considered an investigational technique with potential future clinical value. Texture features are categorized into:

- First-order metrics: Calculated from histogram-based metrics, typically representing the frequency of uptake intensity values.
- Second-order metrics: Derived from the Gray-Level Co-Occurrence Matrix (GLCM), assessing the spatial relationships between pixel intensities.
- Higher-order metrics: Include metrics like the Neighborhood Gray-Tone Difference Matrix (NGTM), which measures differences between adjacent voxels, the Gray-Level Run-Length Matrix (GRLM), which considers the length of runs of identical intensity, and the Gray-Level Size Zone Matrix (GLSZM), which evaluates the size of contiguous voxel zones with the same intensity [67–69].
  Texture analysis is influenced by various factors such as VOI size, software implementation, scanner type, image reconstruction methods, and histogram binning strategies. Therefore, standardized protocols are necessary to enhance reproducibility and reliability for clinical use [70, 71].
  (4) Reference organ uptake

Quality assessments and quantitative analyses often measure uptake in reference organs, such as the mediastinal blood pool or liver.

- Liver: A spherical VOI (3 cm diameter) is placed in the right lobe, avoiding malignancies or boundary effects. Average SUL values range from 1.0 to 2.2 (SUV: 1.3 to 3.0).
- Mediastinal blood pool: VOIs are drawn within the thoracic aorta, ensuring the vessel wall is excluded. Average mediastinal blood pool SUL values are approximately 1.2 (SUV: 1.6).

The values of reference organ uptake may be affected by the equipment, PET/CT protocol or technical issues [72–74].

4) General interpretation criteria

Key considerations for interpreting ^18^F-FDG PET/CT include:

- Clinical imaging purpose and requisition
- Imaging protocols and reconstruction methods (including attenuation correction)
- Physiological ^18^F-FDG distribution and patient-specific variability
- Clinical data, laboratory findings, and other imaging modalities
- Potential false negatives (e.g. small lesions, low-glucose-metabolizing tumors, hyperglycemia, or nearby high physiological uptake)
- Potential false positives (e.g. injection site uptake, attenuation overcorrection, or unrelated pathologies)

Special attention is required for attenuation correction artifacts (e.g. metallic implants). Evaluating both attenuation-corrected and non-corrected images is recommended.

### Reporting

The report is recommended to include the following information and is recommended to provide a concise and structured format that addresses the specific clinical questions posed [75].

1. Basic information

The report includes essential details such as the examination name, patient’s full name, hospital registration number, date and time of examination, as well as optional information such as patient’s gender, age, or date of birth, requesting department, and referring physician.

2) Clinical information

The report describes the clinical purpose of the examination as indicated by the referring physician, along with specific clinical questions to be addressed. This may include diagnostic information, relevant prior treatments, findings from other diagnostic tests, and prior PET/CT results for comparison.

3) Procedure description

The report includes details such as the radiopharmaceutical name, dose (in MBq or mCi), administration route (intravenous), time of administration, and injection site. Additional pretreatment details, such as sedative use, dose, and timing, are also recorded. Imaging specifics, including scan time, scan range, and patient positioning (e.g. supine position, arm placement), are noted, especially if positioning deviates from the standard protocol. Details regarding attenuation correction and any notable issues (e.g. motion artifacts, CT contrast usage) are included.

4) Findings description

Findings are described anatomically and metabolically in relation to the patient’s clinical presentation. Abnormal lesions, their location, ^18^F-FDG uptake patterns, and CT findings are documented. Key findings are prioritized and presented logically, while unexpected abnormalities or potential diagnostic limitations (e.g. small lesions, low-grade malignancies) are explicitly noted. Comparative analysis with prior imaging and quantitative indices, particularly for treatment response evaluation (e.g. Positron Emission Tomography Response Criteria in Solid Tumors [72], Deauville criteria for lymphoma [76–78]), is also provided when relevant.

5) Conclusion

The report highlights the most significant findings and the most likely diagnosis, with differential diagnoses if applicable. Recommendations for follow-up PET/CT or other diagnostic tests may be included if necessary.

## Equipment Specifications

A 3D PET/CT scanner with a low-dose CT for attenuation and scattering correction of PET emission data is recommended. The imaging acquisition system must be capable of collecting static/dynamic or list mode PET emission data in 3D mode and should allow reconstruction of images both before and after attenuation correction in single or multiple frames. Non-attenuation-corrected PET images should not be used for primary interpretation, but may be useful for recognizing artifacts in attenuation-corrected PET images. Additionally, the imaging acquisition system must be capable of online random correction, scattering correction, attenuation correction, dead time correction, and normalization [79]. Digital PET scanners using semiconductor detectors offer advantages such as higher spatial resolution and faster data acquisition and processing compared to traditional PET equipment [80]. As of June 2024, the ratio of digital PET/CT scanners in Republic of Korea was reported to be 34% (*n* = 27 of 79).

## Quality Assurance, Safety, Infection Control, and Patient Education

### Quality Assurance and Safety

To achieve high efficiency and reliability in nuclear medicine imaging examinations, an appropriate quality assurance system, known as quality control, is necessary. Quality control for nuclear medicine imaging equipment is a means of guaranteeing quality management after installation and is essential for improving the quality of care and ensuring patient safety. Each institution must follow maintenance provided by the supplier and appropriate quality control procedures, and examinations for all patients should only proceed if checks have been completed and no abnormalities are detected, adhering to the quality control guidelines. Specific items of quality control should follow the KSNM’s quality control guidelines [81].

### Infection Control

Infection control in this guideline is based on the Infection Control in Medical Institutions (5th edition) published by the Korean Society of Healthcare-associated Infection Control and Prevention [82]. Hand hygiene is performed at every step related to patient contact, and used syringes are disposed of in a needle box without covering the cap. Before the examination, the patient’s infection status is confirmed, and appropriate protective equipment (gloves, masks, plastic aprons, goggles) is worn in cases of confirmed infection. Specific actions depend on the type of infection [82]. Commonly, a sheet should be used to cover the equipment table to reduce contamination from patient contact, and after the examination, an environmental disinfectant officially approved by the regulatory authority should be used to clean the equipment table and any contact surfaces. Contaminated sheets and materials should be separately stored and disposed of properly.

### Patient Education

An examiner explains the following content to patients to alleviate their fears and questions about the examination and encourage their cooperation:

- This examination is performed as an ^18^F-FDG PET/CT examination for diagnosing conditions.
- A minimum fasting period of more than 4 h is required before the examination.
- ^18^F-FDG will be administered intravenously, and patients lie comfortably in the waiting room for approximately 60 min after the injection to allow the drug to distribute well in the body.
- Patients should urinate just before the examination.
- The examination will take about 10–30 min, and there should be no pain involved in the process.
- After the examination, confirmation will be made to ensure it has been successfully completed, and additional imaging may be necessary if required.
- No special measures are needed after the examination.
- If patients have claustrophobia, they inform the medical staff in advance.

## Data Availability

Data sharing not applicable to this article as no datasets were generated or analyzed during the current study.

## Declarations

### Generative AI in Scientific Writing

Authors did not use generative AI in writing except for checking grammar, spelling, references, etc.
