# Correction to: ^18^F-FDG PET/CT for Oncological Patients: Procedural Guideline by the Korean Society of Nuclear Medicine Version 2.0

**DOI:** 10.1007/s13139-025-00942-0

**Published:** 2025-07-31

**Authors:** Eun Ji Han, Chae Hong Lim, Jinkyoung Oh, Joon Young Choi, The Korean Society of Nuclear Medicine Medical Affairs Committee

**Affiliations:** 1 Department of Nuclear Medicine, Yeouido St. Mary’s Hospital, College of Medicine, The Catholic University of Korea, Seoul, Republic of Korea; 2 Department of Nuclear Medicine, Soonchunhyang University Seoul Hospital, Seoul, Republic of Korea; 3 Department of Nuclear Medicine, Incheon St. Mary’s Hospital, College of Medicine, The Catholic University of Korea, Seoul, Republic of Korea; 4 Department of Nuclear Medicine, Samsung Medical Center, Sungkyunkwan University School of Medicine, 81 Irwon-ro, Gangnam-gu, 06351 Seoul, Republic of Korea

**Correction to: Nuclear Medicine and Molecular Imaging**

10.1007/s13139-025-00928-y

The article ^18^F-FDG PET/CT for Oncological Patients: Procedural Guideline by the Korean Society of Nuclear Medicine Version 2.0, written by Eun Ji Han, Chae Hong Lim, Jinkyoung Oh, Joon Young Choi and The Korean Society of Nuclear Medicine Medical Affairs Committee, was originally published under exclusive licence to Korean Society of Nuclear Medicine 2025. As a result of the subsequent decision to publish the article under the open access model, the article’s copyright notice was changed on July 17, 2025 to © The Author(s) (2025) and the article is now distributed under a Creative Commons Attribution (CC BY) license 4.0 International License, which permits use, sharing, adaptation, distribution and reproduction in any medium or format, as long as you give appropriate credit to the original author(s) and the source, provide a link to the Creative Commons licence, and indicate if changes were made.

The images or other third party material in this article are included in the article’s Creative Commons licence, unless indicated otherwise in a credit line to the material. If material is not included in the article’s Creative Commons licence and your intended use is not permitted by statutory regulation or exceeds the permitted use, you will need to obtain permission directly from the copyright holder.

To view a copy of this licence, visit http://creativecommons.org/licenses/by/4.0/.

The original article has been corrected.

